# Bone Marrow Molecular Markers Associated with Relapsed/Refractory Activated B-Cell-Like Diffuse Large B-Cell Lymphoma

**DOI:** 10.1155/2018/1042597

**Published:** 2018-11-11

**Authors:** Di Wang, Peng Liu, Yue Zhang, Hui-Ying Liu, Di Shen, Yi-Qun Che

**Affiliations:** ^1^Department of Clinical Laboratory, National Cancer Center/ National Clinical Research Center for Cancer/ Cancer Hospital, Chinese Academy of Medical Sciences and Peking Union Medical College, Beijing 100021, China; ^2^Department of Medical Oncology, National Cancer Center/ National Clinical Research Center for Cancer/ Cancer Hospital, Chinese Academy of Medical Sciences and Peking Union Medical College, Beijing 100021, China; ^3^Department of Clinical Laboratory, Beijing Chaoyang District Sanhuan Cancer Hospital, Beijing 100122, China

## Abstract

Activated B-cell-like diffuse large B-cell lymphoma (ABC-DLBCL) is a common subtype of non-Hodgkin's lymphoma and is very likely to infiltrate the bone marrow. Over 30% of patients are converted to relapsed/refractory DLBCL after first-line rituximab combined with cyclophosphamide, doxorubicin, vincristine, and prednisone therapy, with a poor prognosis. Our aim was to identify molecular markers that might be utilized to predict relapsed/refractory ABC-DLBCL patients. Hence, we collected bone marrow aspirate smears from 202 patients with ABC-DLBCL and detected expression of bone marrow molecular marker proteins by immunocytochemistry. Signal transducer and activator of transcription (Stat)3, nuclear factor (NF)-*κ*B p65, Syk, Bruton's tyrosine kinase (BTK), and Bcl2 proteins were strongly expressed in bone marrow aspirate smears of ABC-DLBCL patients. The same smear could present positive expression of multiple proteins simultaneously. Positive combinations of protein expression were associated with resistance. The most significant finding was that the Stat3^+^NF-*κ*B^+^ group developed resistance, which was significantly higher than that of the Stat3^−^NF-*κ*B^−^group (80 vs. 14%). There was a significant difference in two-year relapse-free survival between protein-positive and protein-negative combinations of Stat3-NF-*κ*B (P = 0.005), Bcl2-Stat3 (P = 0.009), Bcl2-Pax5 (P = 0.003), and BTK-Syk (P < 0.001). Thus, we detected key molecules in multiple signaling pathways in bone marrow aspirate smears. At the same time, the results provide further clinical evidence of ABC-DLBCL drug-resistant molecules and provide a theoretical basis for rational second-line treatment after drug resistance.

## 1. Introduction

Non-Hodgkin's lymphoma (NHL) is one of the top 10 causes of cancer mortality [1]. Diffuse large B-cell lymphoma (DLBCL) is the most common NHL, accounting for approximately 32.5% of newly diagnosed NHL cases per year. It is a group of malignant tumors with extensive heterogeneity in clinical manifestations, histomorphology, and prognosis [2]. DLBCL can occur in any organ or any part of the body and manifests clinically with an invasive course [3]. According to the different cell origins, DLBCL is divided into activated B-cell-like (ABC)-DLBCL and germinal center B-cell-like (GCB)-DLBCL. The former has a poor prognosis and easily invades the bone marrow [4].

Among the East Asian population, including Chinese, the proportion of ABC-DLBCL is much higher than that of European and American populations. The current first-line treatment for DLBCL is rituximab combined with cyclophosphamide, doxorubicin, vincristine, and prednisone(R-CHOP). The R-CHOP regimen significantly improves outcomes in patients with DLBCL with the overall cure rate reaching approximately 70%. However, with first-line treatment of ABC-DLBCL patients, the long-term progression-free survival rate is less than 50%. Furthermore, among first-line treatment-resistant patients, more than 70% of the conventional second-line regimens cannot achieve complete remission [5]. Although cell origin is currently not used to guide therapy, treatments for ABC-DLBCL and GCB-DLBCL differ in their efficacy due to differences in signaling pathways [6]. Therefore, how to screen patients with relapsed/refractory DLBCL after the R-CHOP-like regimen is an important problem to be solved.

Relapsed lymphoma refers to recurrence three months or more after completing remission (CR). Refractory lymphoma means that any of the following occur: (1) four courses of standardized chemotherapy result in tumor shrinkage < 50% or disease progression; (2) the standard chemotherapy regimen reaches CR but relapses occur within six months; (3) two or more recurrences after CR; or (4) recurrence after hematopoietic stem cell transplantation [7]. Newly diagnosed DLBCL patients require a bone marrow examination to evaluate the staging [8]. At the same time, bone marrow invasion is an independent relapse indicator of DLBCL, and the prognosis following bone marrow invasion is poor [9]. In this study, we collected bone marrow aspirate smears from 202 patients with ABC-DLBCL and screened for refractory-related proteins in ABC-DLBCL-resistant or early relapsed patients through follow-up and immunocytochemistry. The results provide a basis for risk stratification and individualized treatment.

## 2. Materials and Methods

### 2.1. Patients and Clinical Data

We recruited 202 patients with ABC-DLBCL at the National Cancer Center/Cancer Hospital, Chinese Academy of Medical Sciences (Beijing, China), from January 2009 to January 2016. The cohort included 115 males and 87 females with a median age of 49 years (range: 7 to 80 years of age). All patients underwent bone marrow puncture to evaluate staging before R-CHOP treatment and were confirmed to have DLBCL by pathological and imaging examinations. The patients were enrolled according to the following criteria: (i) 6-8 cycles of R-CHOP standard chemotherapy were received and complete clinical data were available; (ii) immunohistochemistry confirmed the presence of ABC-DLBCL; (iii) patients had bone marrow infiltration or minimal bone marrow infiltration by cell morphology and fluorescence in-situ hybridization analysis [10]; and (iv) no other malignant diseases, active hepatitis or diabetes were diagnosed. This retrospective study was approved by the Institutional Review Board of the Cancer Hospital, Chinese Academy of Medical Sciences (Beijing, China). Written informed consent was provided by all patients prior to enrollment in the study.

### 2.2. Bone Marrow Aspiration

Employing standard techniques, approximately 0.5 mL of bone marrow aspirate was taken from the posterior superior iliac spine using a disposable bone marrow aspiration package (Hanaco, Tianjin, China). Each case had at least six smears, and we ensured that bone marrow smears were labeled correctly and clearly. Blood smears were performed using the International Council for Standardization in Hematology recommendation of EDTA-K2 at a concentration of 1.50 ± 0.25 mg/mL of peripheral blood. Each air-dried smear was stained with Wright and Giemsa stains (Baso, Zhuhai, China) for 15 min. Each bone marrow aspirate smear was examined for morphological details and evaluated by two pathologists.

### 2.3. Immunocytochemistry Analysis

Bone marrow aspirate smears were removed from storage at -20°C and equilibrated at room temperature for 30 min and then dewaxed and rehydrated with a graded ethanol series. To quench endogenous peroxidase activity, sections were incubated with 1% H_2_O_2_ at room temperature for 10 min. For antigen retrieval, sections were heated in Tris-EDTA (PH9.0) for 20 min using a microwave oven. The slides were blocked with normal goat serum (ready-to-use; cat. number ZLI-9022; OriGene Technologies, Beijing, China) at 37°C for 1 h. Samples were then incubated with the primary antibodies overnight at 4°C. Incubation with the secondary antibodies and staining were performed using an immunohistochemical staining kit (cat. number PV-9000; OriGene Technologies) according to the manufacturer's protocol. The immunoreaction was visualized with diaminobenzidine staining, followed by counterstaining with hematoxylin. The slides were dehydrated in gradient ethanol and placed in xylene for transparency and then sealed and examined microscopically. Percentages of positively stained tumor cells and staining intensity were evaluated independently by two experienced experts who were blinded to the clinical information. When positive lymphoma cells occurred or positive lymphocytes accounted for ≥ 5% of the total number of lymphocytes, the protein was considered to be overexpressed and defined as “+”. A “2+” rating refers to the presence of positive lymphoma cells or positive lymphocytes accounting for ≥ 10% of the total number of lymphocytes.

The primary antibodies were as follows: rabbit monoclonal anti-Stat3 (dilution, 1:500; cat. number 12640); rabbit monoclonal anti-NF-*κ*B p65 (dilution, 1:00; cat. number ab16502); rabbit monoclonal anti-Syk (dilution, 1:50; cat. number ab40781); rabbit monoclonal anti-BTK (dilution, 1:50; cat. number ab208937); rabbit monoclonal anti-Bcl2 (dilution, 1:100; cat. number ab32124); rabbit monoclonal anti-Pax5 (dilution, 1:100; cat. number ab109443); rabbit monoclonal anti-Bcl6 (dilution, 1:100; cat. number 14895); rabbit monoclonal anti-p57 Kip2 (dilution, 1:100; cat. number 2557); and rabbit monoclonal anti-c-myc (dilution, 1:50; cat. number #9402); Anti-Stat3, Bcl6, P57KIP2, and Pax5 were from Cell Signaling Technology (Danvers, MA, USA). All other antibodies were from Abcam (Cambridge, UK).

### 2.4. Follow-Up

All patients were evaluated radiographically or by positron emission tomography-computed tomography at 2, 4, 6, and 8 cycles of chemotherapy. After completing standard first-line chemotherapy, they were reviewed regularly every three months to determine whether recurrence or progression of the cancer occurred. All patients were followed until recurrence or the end of this study on January 31, 2016. Relapse-free survival (RFS) was defined as the time from the beginning of chemotherapy to the identification of metastasis, or until the last follow-up.

### 2.5. Statistical Analysis

SPSS version 22.0 software (IBM, Chicago, IL, USA) was used for statistical analysis. Discrete variables were compared using Fisher's exact and *χ*2 tests. P ≤ 0.05 was considered statistically significant.

## 3. Results

### 3.1. Positive Rates of Stat3, NF-*κ*B p65, Bcl2, Syk, and BTK Proteins in Patients with ABC-DLBCL

**Figure 1:**
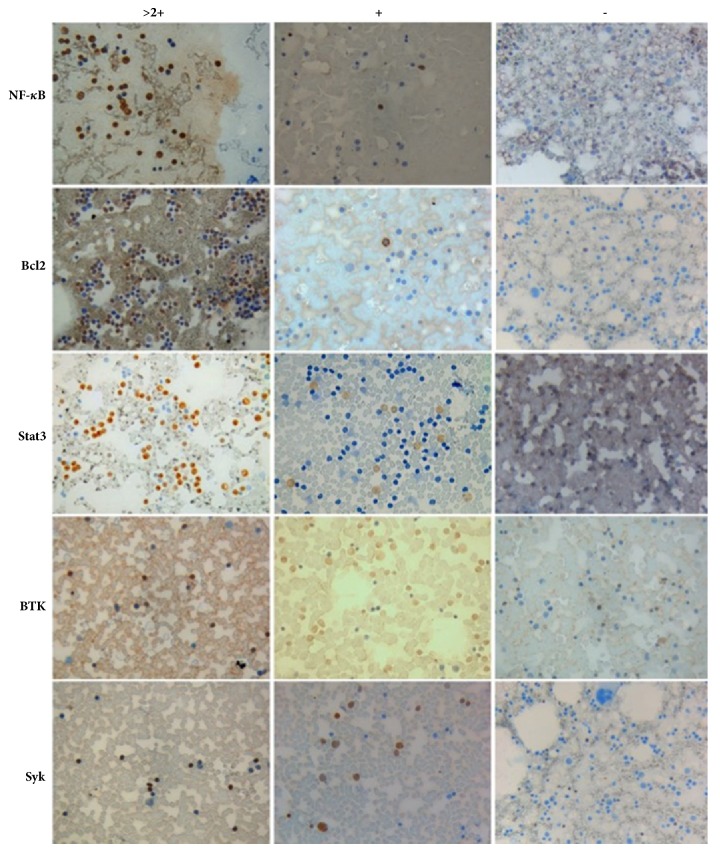
Protein expression of NF-*κ*B p65, Stat3, Syk, BTK, and Bcl2 in bone marrow aspirate smears (×400).

We analyzed expressions of the NF-*κ*B p65, Stat3, Bcl2, Syk, BTK, Pax5, Bcl6, and c-myc proteins in bone marrow cells of 202 patients with ABC-DLBCL by immunocytochemistry. Proteins with higher expression levels were Stat3 (46.0%), NF-*κ*B p65 (38.6%), Syk (38.1%), BTK (28.2%), Bcl2 (23.3%), and Pax5 (21.2%). The positive rates of Bcl6, P57KIP2, and c-myc proteins were low (Table 1). The proteins strongly expressed in bone marrow smears were Stat3, NF-*κ*B p65, Syk, BTK, and Bcl2 (Figure 1).

### 3.2. Bone Marrow Smears from the Same Case Can Express Multiple Proteins Simultaneously

**Figure 2:**
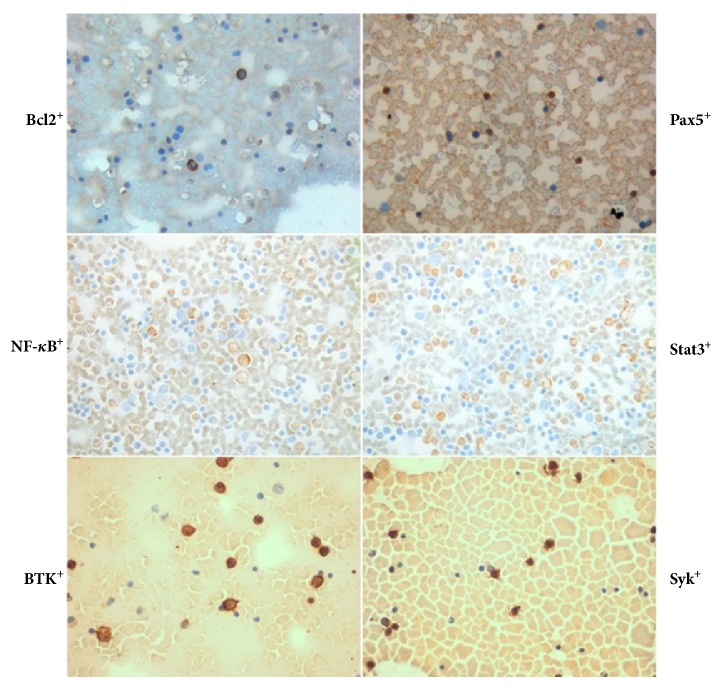
Combined expression of NF-*κ*B^+^Stat3^+^, BTK^+^Syk^+^, and Bcl2^+^Pax5^+^ proteins in the same smear sample (×400).

The expression of multiple proteins in bone marrow aspirate smears of 202 ABC-DLBCL patients was detected by immunocytochemistry. Intriguingly, two of the NF-*κ*B p65, Stat3, Bcl2, Pax5, Syk, and BTK proteins were expressed in numerous smear samples (Figure 2).

### 3.3. Positive Combinations of Protein Expression Are Associated with Drug Resistance of ABC-DLBCL Patients

**Figure 3:**
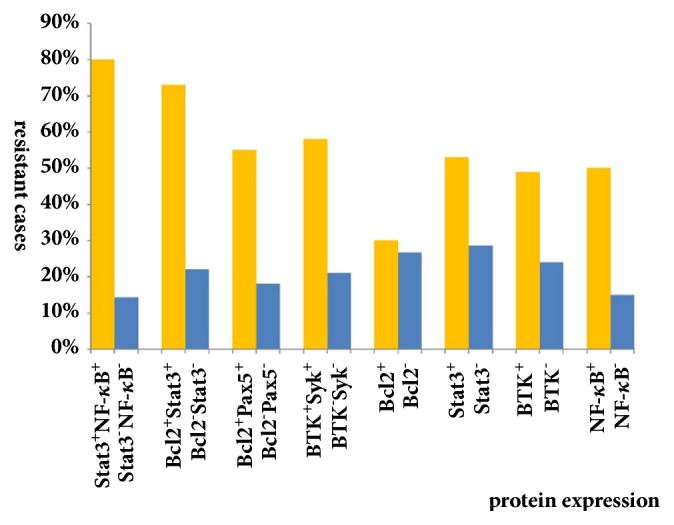
Patients exhibiting drug resistance when smears have positive combinations of protein expression.

Analysis of 202 patients with ABC-DLBCL showed that there were significantly more drug-resistant cases in those with positive combinations of protein expression than those without such combinations. The most significant difference was that the Stat3^+^NF-*κ*B^+^ group developed significantly more resistance than the Stat3^−^NF-*κ*B^−^group (80* vs.* 14%). Comparing the ratio of drug-resistant cases in each group, the difference between the double-positive or double-negative groups was significantly greater than the difference between samples with single protein expression (Figure 3).

### 3.4. Positive Combinations of Protein Expression Are Associated with Recurrence of Cancer in ABC-DLBCL Patients

The relationship between the expression of two protein combinations and two-year RFS of ABC-DLBCL patients was analyzed. There were significant differences in the two-year RFS rates between double-positive and double-negative protein expression groups of Stat3-NF-*κ*B (P = 0.005), Bcl2-Stat3 (P = 0.009), Bcl2-Pax5 (P = 0.003), and BTK-Syk (P < 0.001) (Table 2).

## 4. Discussion

Emergence of the R-CHOP-like cancer treatment regimen has significantly improved overall and progression-free survival of DLBCL patients, with about 70% of patients being cured by chemotherapy, radiotherapy, and immunotherapy. However, 30% of patients still relapse or develop refractory tumors after the initial treatment [11]. Patients with relapsed/refractory DLBCL have only about a 10% chance of a cure, even with high-dose chemotherapy combined with autologous hematopoietic stem cell transplantation [5]. These patients are the leading cause of death among those with DLBCL.

With the recently improved understanding of the molecular pathogenesis of DLBCL subtypes in recent years, the World Health Organization requires identification of DLBCL not otherwise specified subtypes based on cell origin and gene expression profiles,* that is*, GCB and ABC types [12]. These two types differ in terms of chromosomal changes, signaling pathway activation, and clinical outcomes. GCB-DLBCL is characterized by persistent somatic hypermutation, up-regulation of BCL6, and near-universal CD10 expression. ABC-DLBCL is associated with chronic activated B-cell receptor (BCR) signaling and NF-*κ*B dysregulation [13]. The Chinese population has more ABC-DLBCL than GCB-DLBCL patients, whose prognosis is worse causing widespread concern.

In the R-CHOP-like regimen, rituximab inhibits the NF-*κ*B pathway through the CD20 receptor and induces apoptosis or increases the sensitivity of drug-resistant cells, thereby achieving positive treatment outcomes. Abnormal activation of the NF-*κ*B signaling pathway and overexpression of downstream antiapoptotic proteins such as Bcl-2, Bcl-xL, and Mcl-1 may be the main causes of drug-resistant ABC-DLBCL [14].

Genome-wide analysis can confirm individual somatic mutations or transcript abnormalities, but genetic alterations alone are insufficient to trigger ABC-DLBCL resistance. Mutations or aberrant activation of some key genes result in the inability of candidate target inhibitors to participate in the NF-*κ*B signaling pathway. Though intracellular NF-*κ*B interacts with multiple important pathways, many molecules closely related to the tumor activate the NF-*κ*B pathway. Rituximab can inhibit the p38 MAPK signaling pathway, disrupt the circulating secretion of IL-10/IL-10 receptor, inhibit the Stat3 pathway, and downregulate the antiapoptotic molecule, Bcl2. Thus, it is speculated that increased expression of Stat3 may be an important cause of rituximab resistance [15].

BTK is an intermediate key signal between BCR and IKK and is involved in the NF-*κ*B antiapoptotic pathway [16]. The BTK inhibitor, ibrutinib, is a key target for the BCR signaling pathway of ABC-DLBCL [17]. BCR signaling in ABC-DLBCL cells activates SYK in the phosphoinositide 3-kinase pathway, and SYK inhibitors cooperate with ibrutinib to kill ABC-DLBCL cells [18]. In our preliminary work, high expression of the Bcl2 protein was also found to cause refractory DLBCL. Thus, patients with Bcl2 gene translocation and amplification have a poor prognosis [10]. Accurate detection of Bcl2 expression is important for both the prognosis and the Bcl2 antibody treatment of patients with DLBCL [19].

According to the above research, our team screened for a group of highly expressed refractory-related proteins in relapsed/refractory ABC-DLBCL patients including NF-*κ*B p65, Stat3, Bcl2, Syk, BTK, Pax5, Bcl6, c-myc, and P57KIP2 by tissue chip. However, the tissue obtained by puncturing from many new patients for diagnosis is too small to perform immunohistochemical analysis of various proteins. Also, most people undergo bone marrow aspiration to assess staging. We studied expression of these proteins in bone marrow aspirate smears and analyzed their relationship with drug resistance and recurrence. Thus, we not only used minimum bone marrow (a bone marrow puncture test is 0.5 mL) to identify the staging of the bone marrow infiltration but also identified the relapsed/refractory-related proteins without an additional burden on the patients.

Our results showed that the Stat3, NF-*κ*B p65, Syk, BTK, and Bcl2 proteins were strongly expressed in 202 ABC-DLBCL bone marrow smears, and the same smear could exhibit positive expression of two proteins at the same time. The number of cases expressing several proteins was less than 202 because of the occurrence of detachments during immunocytochemistry. After follow-up, our statistical analysis found that ABC-DLBCL patients expressing a combination of proteins were significantly more likely to exhibit tumor resistance and poorer two-year RFS rates. This suggests that core molecular aberrant protein combinations of the NF-*κ*B pathway are associated with relapse/refractory ABC-DLBCL after R-CHOP-like treatment. This is the first report that the condition of this pathway in the bone marrow is directly related to prognosis.

In conclusion, gene expression analysis enhances our understanding of the molecular mechanisms of chemotherapeutic drug resistance. The emergence of cross-resistance requires identification of a rational target for the treatment of refractory ABC-DLBCL. Acquired resistance is driven by inherent genetic heterogeneity and tumor cell instability. Due to the heterogeneity of DLBCL, multiple pathways lead to drug resistance, and the treatment of DLBCL requires drug combinations targeted to multiple pathways [20]. This study identified molecular markers that might be used to predict patients with relapsed/refractory ABC-DLBCL by using bone marrow aspiration smears to detect key molecules in multiple signaling pathways in which drug resistance occurs. At the same time, the results will further enrich the clinical evidence of ABC-DLBCL drug-resistant molecules and provide a theoretical basis for rational second-line treatment after drug resistance.

## Figures and Tables

**Table 1:** Protein expression of bone marrow cells from patients with activated B-cell-like diffuse large B-cell lymphoma.

Proteins	Bone marrow positive rates	Localization
Stat3	46.0% (93/202)	nucleus
NF-*κ*B p65	38.6% (78/202)	nucleus
Syk	38.1% (77/202)	nucleus
BTK	28.2% (57/202)	nucleus
Bcl2	23.3% (47/202)	cytoplasm
Pax5	21.2% (33/156)	nucleus
Bcl6	15.4% (16/104)	nucleus
P57KIP2	3.8% (4/104)	nucleus
c-myc	7.7% (8/104)	nucleus

**Table 2:** The relationship between protein combinations and relapse-free survival of ABC-DLBCL patients.

Protein combinations	Relapse	Nonrelapse	*χ* ^2^	P
Stat3^+^NF-*κ*B^+^	30	18	7.737	0.005
Stat3^*-*^NF-*κ*B^*-*^	39	63		
Bcl2^+^Pax5^+^	10	21	8.846	0.003
Bcl2^*-*^Pax5^*-*^	65	39		
Bcl2^+^Stat3^+^	26	14	6.778	0.009
Bcl2^*-*^Stat3^*-*^	42	61		
BTK^+^Syk^+^	33	20	16.670	4.4 × 10^−5^
BTK^*-*^Syk^*-*^	20	56		

## Data Availability

The data used to support the findings of this study are available from the corresponding author upon request.

## Abbreviations

ABC-DLBCL: Activated B-cell-like diffuse large B-cell lymphoma
NHL: Non-Hodgkin's lymphoma
RR-DLBCL: Relapsed/refractory DLBCL
R-CHOP: Rituximab combined with cyclophosphamide, doxorubicin, vincristine, and prednisone
Stat3: Signal transducer and activator of transcription 3
NF-*κ*B p65: Nuclear factor-*κ*B p65
Syk: Spleen tyrosine kinase
BTK: Bruton's tyrosine kinase
Bcl6: B-cell lymphoma-6
Bcl2: B-cell lymphoma-2.
